# Understanding youths’ attitudes and practices regarding listening to music, video recording and terrain park use while skiing and snowboarding

**DOI:** 10.1186/s12887-020-02292-6

**Published:** 2020-08-19

**Authors:** Kelly Russell, Stephanie Arthur, Claude Goulet, Erin Selci, Barbara Morrongiello

**Affiliations:** 1 Department of Pediatrics and Child Health, Max Rady College of Medicine, Rady Faculty of Science, CE-208 Children’s Hospital, 840 Sherbrook Street, Health Sciences Center, Winnipeg, Manitoba R3A 1S1 Canada; 2 Children’s Hospital Research Institute of Manitoba, John Buhler Research Centre, 513-715 McDermot Avenue, Winnipeg, MB R3E 3P4 Canada; 3 Department of Physical Education, Laval University, Pavillion des Sciences de l’education, 2320, rue des Bibiotheques, Quebec City, Quebec G1V 0A6 Canada; 4 Department of Psychology, University of Guelph, 50 Stone Road East, Guelph, Ontario N1G 2W1 Canada

**Keywords:** Skiing, Snowboarding, Behaviours, Youth, Music, Terrain parks, Video recording

## Abstract

**Background:**

Skiing and snowboarding are popular activities among Canadian youth and these sports have evolved to include certain risk behaviours such as listening to music, using terrain parks, and video recording yourself or others. The objective of this study was to determine the prevalence of these risk behaviours and identify factors that are associated with the risk behaviours.

**Methods:**

Using focus group methodology, a questionnaire was developed to capture aspects of the Theory of Planned Behaviour. A cross-sectional study was conducted where the questionnaire was administered to youth aged 13–18 during two winter seasons at two ski hills in Manitoba, Canada.

**Results:**

The sample was comprised of 735 youth (mean age 14.9; 82.1% male, 83.6% snowboarding). The most common behavior was using the TP (83.1%), followed by listening to music that day (36.9%), and video recording that day (34.5%). Youth had significantly higher odds of listening to music that day if they planned to next time (OR 19.13; 95% CI: 10.62, 34.44), were skiing or snowboarding alone (OR 2.33; 95% CI: 1.10, 4.95), or thought listening to music makes skiing or snowboarding more exciting or fun or makes them feel more confident (OR 2.30; 95% CI: 1.31, 4.05). They were less likely to if they believed that music made it more difficult to hear or talk to others (OR: 0.35; 95% CI: 0.18, 0.65). Youth had significantly higher odds of using the terrain park if they believed that terrain parks were cool, challenging, or fun (OR: 5.84; 95% CI: 2.85, 11.96) or if their siblings used terrain parks (OR: 4.94; OR: 2.84, 9.85). Those who believed that terrain parks were too busy or crowded (OR: 0.31; 95% CI: 0.16, 0.62) were less likely to use them. Youth had significantly higher odds of video recording that day if they reported that they plan to video record next time (OR: 8.09, 95% CI: 4.67, 14.01) or if they were skiing or snowboarding with friends (OR: 3.65, 95% CI: 1.45, 9.18). Youth had significantly higher odds of video recording that day if they agreed that recording makes them try harder and improved their tricks (OR: 3.34, 95% CI: 1.38, 8.08) compared to those who neither agreed nor disagreed. Youth were less likely to record themselves that day if their friends did not do so (OR: 0.36; 95% CI: 0.16, 0.80).

**Conclusion:**

Common predictors of engaging in risk behaviours suggest that injury prevention programs may not have to be specific to each behaviour. Some strategies for injury prevention are suggested.

## Background

Skiing and snowboarding remain popular winter sports in Canada, with over a million people participating in at least one of these activities every year [1]. Approximately 5 % of households in Canada with youth aged 13–19 participated in skiing, snowboarding, or telemarking (i.e., a type of downhill skiing) close to their home in 2015 [2]. Skiing and snowboarding have evolved with the introduction of terrain parks (TPs) and new equipment. For example, skis and poles are sold specifically for the execution of aerial and non-aerial manoeuvres, helmets now contain built in speakers for ease of listening to a personal music player, and helmets can be mounted with GoPro cameras to capture ski and snowboard runs and tricks.

Sport and recreation are common causes of unintentional injury, especially among youth [3–5]. Adolescents often ski and snowboard with friends and seize the opportunity to engage in high-risk behaviours, often judging themselves to be impervious to injury [6–8]. The overall injury risk is estimated to be 2–4 injuries per 1000 participant days [9–13] but is higher among 7–17 year olds [14]. Injuries or the potential for injuries have been linked to a variety of high-risk behaviours associated with skiing and snowboarding among youth. For example, injuries sustained in TPs, which are commonly used by youth, are more severe than those occurring on the regular slope [15]. In addition, the proportion of injuries occurring in terrain parks has also increased over time [16]. Snowboarders who listen to music in TPs have a higher risk of severe injury [17]. Also, listening to music while wearing a ski or snowboard helmet results in reduced sound source localization [18] and this inability to locate sounds within a dynamic slope may increase injury risk. Video recording may increase the risk of injuries if the skier or snowboarder attempts to ski or snowboard beyond their ability because they are being filmed.

The risk of a ski or snowboard injury can be modified by engaging in safe behaviours [19]. For adolescents, choices between risky or safe behaviour are more frequently made away from the home and in the presence of peers [20, 21]. The decision to engage in a risky behaviour is multifaceted and involves many psychosocial factors [22–24]. For example, decisions are shaped by the individual’s beliefs of injury vulnerability and perceptions of injury severity [25, 26]. Among adolescent skiers and snowboarders, a sense of accomplishment has been shown to be associated with increased safety behaviour, whereas relieving negative emotions is associated with fewer safety behaviours [27]. Parental practices and attitudes towards injury risk can also positively or negatively influence child’s risk taking behaviour [28]. Finally, peers can shift norms, attitudes, values, and perceptions about high-risk behaviours often through verbal persuasion [29] and/or by modelling such behaviours [30].

There is minimal information concerning how youth make decisions to engage in high-risk behaviours while skiing and snowboarding and who or what factors influences these decisions. Before the psychological determinants of behaviour (i.e., attitudes and beliefs) can be changed, a thorough understanding of these factors and how they come into play to impact behaviour is first needed. This has limited the development of broader interventions that address the psychosocial determinants of youth risk behaviours when skiing and snowboarding. Understanding these psychosocial determinants will inform the development of effective strategies to promote safe skiing and snowboarding that can be targeted towards those skiers and snowboarders who engage in high-risk behaviours. Policy makers can then intervene to reduce unsafe behaviour and ideally reduce injuries. The current study addressed this gap. Owing to the considerable evidence that the constructs outlined in the Theory of Planned Behaviour (attitudes, subjective norms, behavioural control) show good prospective prediction of health related behaviours [31], this framework was applied. The objectives of this study were:

1. Determine the prevalence of high-risk behaviours (listening to music, using the TP, and video recording) of youth while skiing and snowboarding,
2. Identify the psychosocial predictors (i.e., *attitudes* toward high-risk behaviours, *perceived subjective norms* about these risk practices, and *perceived behavioural control*) that determine youths’ adoption or the intention to adopt the high-risk behaviours.

## Methods

### Questionnaire development

A questionnaire was developed specifically for this research and the development process followed the nine steps outlined by Francis et al. [23] Six focus groups consisting of 4–10 youth between the ages of 13–18 years who regularly ski and/or snowboard were conducted. The youth were recruited from two ski resort lodges and offered a $25 gift card to participate. To guide the process, youth were asked what they thought of ‘high-risk’ behaviours (i.e., increases risk of injury) while skiing and snowboarding and then discussed what they thought and believed about engaging in these behaviours (why or why not, when might they, benefits to costs, etc.). We then asked: “*What do you think are some of the reasons why some skiers and snowboarders your age choose to use the terrain park while skiing and snowboarding?*” We also asked: *“What behaviours do you think increase your risk of injury when skiing or snowboarding?”*

The focus groups were rooted within the Theory of Planned Behaviour (TPB). For *behaviour*, skiers and snowboarders were asked about current high-risk behaviours and frequency of these behaviours; the theory proposes that intentions are good proxy indices for readiness to perform a behaviour. *Generalised intention* was assessed by asking questions about their willingness to engage in high-risk behaviours the next time they ski or snowboard. *Attitudes towards the behaviour* was determined by assessing both instrumental (behaviour achieves something) and experiential (how it feels to perform the behaviour) attitudes and reflects the extent of which performance of the behaviour is negatively or positively valued. *Subjective norms* were also elicited by reporting sources of social pressure and significant others whose opinions they value. We elicited responses by asking open ended questions about their attitudes towards high-risk behaviours and their perceptions of risk and consequences of engaging or not engaging in the behaviours. *Perceived behavioural control* was assessed by asking about the extent of confidence one has in the ability to adopt or avoid high-risk behaviours. A content analysis was performed and then independently corroborated by a second research assistant (RA) to identify and label themes. The most commonly identified beliefs were transformed into a set of statements or questions that affect engaging in high-risk behaviours and included in the questionnaire. The questionnaire items included what the important people think the youth should do and what the important people actually do.

### Assessing questionnaire validity and reliability

Face validity was assessed by asking 18 psychology students and fellows to assign each item to 1 of 3 categories: attitudes, subjective norms, behavioural control. Items that were consistently assigned (≥ 85% agreement) were retained and the questionnaire was tested among 96 youth at the ski resorts. The youth were reimbursed with a $10 gift card. For validity assessment, the youth were also asked to complete two scales measuring sensation seeking [32, 33] and risk taking propensity [34]. The construct and discriminant validity of the questionnaire was assessed by examining the inter-correlation matrix. Internal reliability was assessed by calculating Cronbach’s alpha. The factor structure was assessed by applying a Confirmatory Factor Analysis to the data to determine which variables could be removed. Finally, psychology students were asked to identify what components of the TPB was being assessed by each question.

### Setting

The focus groups and questionnaires were conducted at two resorts near Winnipeg, Manitoba. Both resorts included TPs that had a variety of features including boxes, rails, jumps, and table tops. One resort had two terrain parks and 50% of the other runs were classified as beginner, 33% as intermediate, and 17% as advanced. The other resort had one terrain park and 50% of the runs were beginner, 25% were intermediate and 25% were advanced. There were no notable changes to the resorts during the study period. Neither resort included any treed/glade runs. The first questionnaire was developed during the 2013–2014 winter season and the final questionnaire (please see Additional file 1 - Questionnaire) was administered during the 2014–2015 and 2015–2016 seasons.

### Participants

English-speaking skiers and snowboarders aged 13–18 years were included. Those who were at the resort but not skiing or snowboarding or had previously completed the questionnaire were excluded.

### Study design

We used a cross-sectional study design to determine the prevalence of youth who engaged in high-risk behaviours and to examine psychosocial predictors of such behaviours and if these predictors differ by other factors. For each of the three high-risk behaviours, youth were classified as having taken part in that specific behaviour (i.e., using TPs, listening to music that day, or video recording that day) or not engaging in the high-risk behaviour.

### Recruitment methods

The season was divided into weekday evening (16:00–22:00), and weekend (Friday 16:00-Sunday 17:00). Data collection occurred in 4 hour periods. Throughout the course of the season, each 4 hour time slot was sampled 3–4 times. If the resort was closed due to inclement weather, data collection was rescheduled to the following week on the same day and time. We have successfully employed this sampling methodology with snowboarders [35]. Two RAs recruited skiers and snowboarders inside the resort lodge at the assigned days and times. Youth were asked to participate in a questionnaire about skiing and snowboarding behaviours and asked if they have previously participated. Youth who stated that they had already participated were excluded. The questionnaire was administered after verbal consent. The youth completed the survey at a table in the resort away from their friends and family. The youth returned the completed survey to the RA, who put in a sealable envelope. Upon completion, the skier or snowboarder received a $10 gift card. Sex and approximate age were visually assessed for those who did not consent. We have previously successfully estimated approximate age in snowboarders [35].

### Outcome and psychosocial exposure assessment

There were three outcomes of interest: listening to music while skiing or snowboarding, using TPs, or video recording while skiing or snowboarding on the day of survey completion. They were asked if they used a TP and why, plan to the next time, what they would do if their parents forbade it, if they thought TPs increased the risk of injury, if their friends, parents or siblings used the TP, and why they thought others used the TP. Youth were asked if they listened to music on the day of survey completion and the listening mechanism: one or two earbuds or helmets with internal speakers. They were asked why they did or did not listen to music and why they believed others did or did not while skiing or snowboarding, if their friends and family also did, and if they intended to do so next time. Finally, youth were asked if they recorded themselves or others on the day of survey competition and if so, what type of recording device they used. They were asked why they did or did not record themselves, if their friends and family recorded themselves while skiing or snowboarding, and if they intended to film themselves or others next time. The youth were also asked to report demographics (age, sex, previous injury, who they were skiing or snowboarding with, and if they anticipated getting hurt today). The questionnaire with TPB classifications is available upon request.

### Sample size and analysis

#### Sample size

The sample size was calculated based on Objective 2: determining psychosocial predictors for high-risk behaviours. Because we were assessing multiple high-risk behaviours, which likely have different rates of engagement among youth, sample size was estimated for a high and low rate of engaging in the behaviour. For example, using listening to music as the high-risk behaviour, it was assumed 10% of those without any psychosocial predictors would listen to music and those with a predictor would have a twofold increase in the odds of listening to music. If alpha is 0.05 and power is 80%, 205 participants with a predictor and 410 with no predictor would be needed for a total of 615 youth. Conversely, if 80% of those without any psychosocial predictors listened to music and the remaining parameters were held the same, we would need 201 youth with a predictor and 401 youth with no predictor for a total of 602 youth.

#### Analysis

The proportion (with 95% CI) of skiers and snowboarders who report each specific high-risk behaviour was calculated. Proportions were stratified by age group, sex, and activity. Baseline characteristics and psychosocial predictors were expressed as proportions and 95% CIs for categorical data and means with standard deviations for continuous data.

Multivariable logistic regression models were built using a forward model building approach as described by Hosmer, Lemeshow, and Sturdivant [36]. Separate models were made for each risk behaviour outcome (listening to music while skiing or snowboarding that day, using the TP while skiing or snowboarding that day, or recording yourself or others while skiing or snowboarding that day). Potential exposure variables included demographic characteristics, perceived risk of personal injury, behaviours and intentions regarding the risk behaviour, friends, parents, and siblings risk behaviour habits, and reasons why youth engage in risk behaviours. Univariate analyses were done using logistic regression for continuous variables and chi-square tests for categorical variables. A cut-off of *p* < 0.20 was used to identify variables for initial inclusion. Variables with low variation (≥ 90% of observations in one category), chi squared expected cell counts ≤5, or high levels of missing values (≥ 50% missing) were excluded. Categorical variables with a low number of responses in one or more category were collapsed. In the analysis of TP usage, a portion of multilevel variables were reduced to dichotomous variables where neither was combined with the agree category as this produced the most precise effect estimates.

A correlation matrix was used to identify potentially redundant variables and evidence of multicollinearity. Those with high correlation (*r* > 0.5) were either combined to create a new variable or one variable was chosen for initial inclusion [37]. A full multivariable model was created and variables with *p*-value < 0.05 in the full model were retained to create a reduced model. Variables with *p* < 0.05 that retained low cell counts after being collapsed and those that produced extremely imprecise estimates, as indicated by wide 95% confidence intervals, were excluded from the analysis. The estimates from the full and reduced models were compared to determine if any of the non-significant variables confounded the estimates in the reduced model. Confounding was defined as a change in odds ratio (OR) by > 10% [38]. If confounding was present, non-significant variables from the full model were re-added one at a time to determine which caused the percent change in the estimate. All confounders were retained in the model.

Any variables that were excluded prior to fitting the full model were then independently added to the model containing significant risk factors and confounders to determine if they were significant at *p* ≤ 0.05. Any variables that became significant were retained in the model, creating the final model [36]. Model fit was assessed by identifying any influential observations and calculating variance inflation factors to determine if collinearity was present.

### Ethical approval

This study received ethical approval from the University of Manitoba – Health Research Ethics Board (Bannatyne Campus).

## Results

### Survey validity and reliability

Among the 18 psychology students who assessed face validity by assigning components of TPB to each survey question, percent agreements ranged from 52 to 100% for music and video recording, and 56–100% for TP usage. Items with low percent agreement (< 85%) were removed from the survey. One (5%) question from the music, 13 (27%) questions from the terrain park, and five (28%) questions from the video recording portion of the survey were removed. Overall, 96 skiing and snowboarding youth completed the initial proposed survey (mean age 15.3 SD: 1.3; 74.0% male, 67.7% snowboarding). All three behaviours were positively correlated with sensation seeking (listening to music: 0.09, TP: 0.08, video recording 0.04) and risk taking (listening to music: 0.24, TP: 0.27, video recording 0.17). For both music and TPs, five factors were identified using confirmatory factor analysis and any survey questions that did not belong in any factor (Cronbach’s alpha < 0.35) were removed from the final questionnaire. For video recording, only two factors were identified and questions that had a Cronbach’s alpha below 0.37 were removed. The remaining results pertain to youth who completed the final and shorter version of the survey. The Flesch-Kincaid reading level was appropriate (grade 4.9).

### Sample characteristics

Overall, 753 youth participated in the study (87% consented to participate); however 18 youth were subsequently excluded due to missing age data (*n* = 11), reporting their age as over 18 (*n* = 2), or reporting their age as 12 (*n* = 5), leaving a total sample size of 735. No youth were excluded because they did not speak English. The mean age of participants was 14.9 years (SD: 1.5). The sample was 82.1% male, 83.6% snowboarders, 11.5% considered themselves beginners, 40.2% as intermediate, 33.8% as advanced, and 14.6% as experts. For each behaviour, the sample was further reduced if youth did not answer the main outcome (today I am using the terrain park/listening to music/recording myself or others) or if there were inconsistencies in their responses (Tables 1, 2 and 3). Of the three high-risk behaviours, the most common behaviour was using the TP (83.1%), followed by listening to music while skiing or snowboarding today (36.9%), and lastly video recording while skiing or snowboarding today (34.5%) (Table 4). Among all three behaviours, males and snowboarders were more likely to engage in the behaviour.

**Table 1:** Baseline characteristics for those listening and not listening to music while skiing or snowboarding today (*N* = 723)

	Not listening to music while skiing or snowboarding today *n* = 456	Listening to music while skiing or snowboarding today *n* = 267	OR (95% CI)
Age (mean (sd))	14.7 (1.5)	15.2 (1.4)	1.26 (1.14, 1.40)
Male	356 (78.1)	235 (88.0)	2.18 (1.40, 3.38)
Missing	1 (0.2)	2 (0.7)	
Snowboarders	364 (79.8)	233 (87.3)	1.92 (1.22, 3.02)
Missing	5 (1.1)	5 (1.9)	
Ability^a^			
Beginner	63 (13.8)	15 (5.6)	1.00
Intermediate	206 (45.2)	78 (29.2)	1.59 (0.86, 2.96)
Advanced	140 (30.7)	98 (36.7)	2.94 (1.58, 5.46)
Expert	36 (7.9)	66 (24.7)	7.70 (3.85, 15.42)
Missing	11 (2.4)	10 (3.7)	
Previous ski/snowboard injury that required a doctor	91 (20.0)	96 (36.0)	2.28 (1.61, 3.23)
Missing	54 (11.8)	27 (10.1)	
Skiing or snowboarding alone^a^	34 (7.5)	42 (15.7)	2.31 (1.43, 3.73)
Missing	2 (0.4)	0	
Skiing or snowboarding with friends	390 (85.5)	242 (90.6)	1.66 (0.99, 2.79)
Missing	7 (1.5)	3 (1.1)	
Skiing or snowboarding with parents	42 (9.2)	22 (8.2)	0.87 (0.51, 1.49)
Missing	14 (3.1)	4 (1.5)	
Skiing or snowboarding with siblings	89 (19.5)	60 (22.5)	1.19 (0.82, 1.72)
Missing	8 (1.8)	3 (1.1)	
Think you will get any type of injury today			
Disagree	309 (67.8)	152 (56.9)	1.00
Neither agree or disagree	59 (12.9)	37 (13.9)	1.27 (0.81, 2.01)
Agree	76 (16.7)	73 (27.3)	1.95 (1.34, 2.84)
Missing	12 (2.6)	5 (1.9)	
Think you will get a head injury today			
Disagree	351 (77.0)	191 (71.5)	1.00
Neither agree or disagree	38 (8.3)	37 (13.9)	1.79 (1.10, 2.91)
Agree	56 (12.3)	34 (12.7)	1.12 (0.70, 1.77)
Missing	11 (2.4)	5 (1.9)	
Think you will get a wrist injury today			
Disagree	316 (69.3)	172 (64.4)	1.00
Neither agree or disagree	50 (11.0)	33 (12.4)	1.21 (0.75, 1.95)
Agree	83 (18.2)	59 (22.1)	1.31 (0.89, 1.91)
Missing	7 (1.5)	3 (1.1)	
I listen to music on my iPod/phone while skiing or snowboarding			
Hardly ever	361 (79.2)	24 (9.0)	1.00
Half the time	70 (15.4)	66 (24.7)	14.18 (8.33, 24.16)
Most of the time	25 (5.5)	175 (65.5)	105.29 (58.45, 189.66)
Missing	0	2 (0.7)	
I plan to listen to music next time^a^	109 (23.9)	233 (87.3)	37.13 (22.18, 62.16)
Missing	17 (3.7)	15 (5.6)	
I want to listen to my own music	216 (47.4)	223 (83.5)	5.76 (3.96, 8.38)
Missing	0	1 (0.4)	
I don’t like the music playing overhead^a^	123 (27.0)	116 (43.4)	2.09 (1.52, 2.88)
Missing	0	1 (0.4)	
Music makes me more aware of my surroundings	43 (9.4)	58 (21.7)	2.68 (1.75, 4.11)
Missing	0	1 (0.4)	
I like being in my own world	132 (28.9)	139 (52.1)	2.69 (1.96, 3.68)
Missing	0	1 (0.4)	
Music makes skiing or snowboarding more exciting or fun or makes them more confident^a^	210 (46.1)	212 (79.4)	4.58 (3.22, 6.51)
Missing	1 (0.2)	1 (0.4)	4.54 (0.28, 73.68)
Parents listen to music while skiing or snowboarding^b^			
No	398 (87.3)	186 (69.7)	1.00
Sometimes	22 (4.8)	23 (8.6)	2.24 (1.22, 4.12)
Yes	18 (3.9)	36 (13.5)	4.28 (2.37, 7.74)
Missing	18 (3.9)	22 (8.2)	
Siblings listen to music while skiing or snowboarding^b^			
No	350 (76.8)	135 (50.6)	1.00
Sometimes	48 (10.5)	44 (16.5)	2.38 (1.51, 3.74)
Yes	38 (8.3)	65 (24.3)	4.43 (2.84, 6.93)
Missing	20 (4.4)	23 (8.6)	
Friends listen to music while skiing or snowboarding			
No	144 (31.6)	24 (9.0)	1.00
Sometimes	164 (36.0)	70 (26.2)	2.56 (1.53, 4.29)
Yes	112 (24.6)	159 (59.6)	8.52 (5.19, 13.98)
Missing	36 (7.9)	14 (5.2)	
Listening to music makes me a better skier or snowboarder^a^			
Disagree	183 (40.1)	54 (20.2)	1.00
Neither agree or disagree	115 (25.2)	44 (16.5)	1.30 (0.82, 2.06)
Agree	151 (33.1)	168 (62.9)	3.77 (2.59, 5.48)
Missing	7 (1.5)	1 (0.4)	
Listening to music makes me more likely to hurt myself or others			
Disagree	174 (38.2)	135 (50.6)	1.00
Neither agree or disagree	89 (19.5)	55 (20.6)	0.80 (0.53, 1.19)
Agree	185 (40.6)	74 (27.7)	0.52 (0.36, 0.73)
Missing	8 (1.8)	3 (1.1)	
Listening to music is safe if you use one ear bud^a^			
Disagree	159 (34.9)	63 (23.6)	1.00
Neither agree or disagree	82 (18.0)	45 (16.9)	1.39 (0.87, 2.21)
Agree	207 (45.4)	158 (59.2)	1.93 (1.35, 2.76)
Missing	8 (1.8)	1 (0.4)	
Listening to music makes me more careful^a^			
Disagree	255 (55.9)	112 (41.9)	1.00
Neither agree or disagree	99 (21.7)	75 (28.1)	1.72 (1.19, 2.51)
Agree	93 (20.4)	80 (30.0)	1.96 (1.35, 2.84)
Missing	9 (2.0)	0	
Listening to music is distracting^a^			
Disagree	154 (33.8)	161 (60.3)	1.00
Neither agree or disagree	87 (19.1)	43 (16.1)	0.47 (0.31, 0.72)
Agree	207 (45.4)	61 (22.8)	0.28 (0.20, 0.40)
Missing	8 (1.8)	2 (0.7)	
Listening to music makes it harder to hear/talk to people^a^			
Disagree	78 (17.1)	82 (30.7)	1.00
Neither agree or disagree	55 (12.1)	29 (10.9)	0.50 (0.29, 0.87)
Agree	314 (68.9)	153 (57.3)	0.46 (0.32, 0.67)
Missing	9 (2.0)	3 (1.1)	
Listening to music is fun or relaxing			
Disagree	102 (22.4)	40 (15.0)	1.00
Neither agree or disagree	76 (16.7)	24 (9.0)	0.81 (0.45, 1.45)
Agree	271 (59.4)	202 (75.7)	1.90 (1.26, 2.86)
Missing	7 (1.5)	1 (0.4)	
Using the terrain park today	343 (75.2)	225 (84.3)	2.60 (1.59, 4.38)
Missing	18 (3.9)	18 (6.7)	
Video recording while skiing or snowboarding today	125 (27.4)	140 (52.4)	2.96 (2.13, 4.11)
Missing	1 (0.2)	2 (0.7)	

^a^Variables included in the logistic regression model

^b^Parent and siblings were combined to ‘any family member’ in the logistic regression model

**Table 2:** Baseline characteristics for those using and not using a terrain park while skiing or snowboarding today (*N* = 691)

	Not using TP today *n* = 117	Using TP today *n* = 574	OR (95% CI)
Age (years)^a^	15.1 (1.6)	14.8 (1.4)	0.89 (0.78, 1.02)
Male^a^	64 (54.7)	500 (87.1)	5.83 (3.75, 9.06)
Missing	0	3 (0.5)	
Snowboarders^a^	81 (69.2)	483 (84.1)	2.55 (1.61, 4.03)
Missing	1 (0.9)	9 (1.6)	
Ability^a^			
Beginner	36 (30.8)	39 (6.8)	1.00
Intermediate	50 (42.7)	223 (38.9)	4.12 (2.38, 7.11)
Advanced	21 (17.9)	210 (36.6)	9.23 (4.88, 17.46)
Expert	9 (7.7)	83 (14.5)	8.51 (3.74, 19.40)
Missing	1 (0.9)	19 (3.3)	
Previous ski/snowboard injury that required a doctor^a^	11 (9.4)	168 (29.3)	4.08 (2.12, 7.83)
Missing	15 (12.8)	65 (11.3)	
Skiing or snowboarding alone	12 (10.3)	61 (10.6)	1.03 (0.54, 1.99)
Missing	1 (0.9)	1 (0.2)	
Skiing or snowboarding with friends^a^	83 (70.9)	523 (91.1)	4.44 (2.65, 7.43)
Missing	3 (2.6)	7 (1.2)	
Skiing or snowboarding with parents	15 (12.8)	49 (8.5)	0.63 (0.34, 1.16)
Missing	4 (3.4)	15 (2.6)	
Skiing or snowboarding with siblings	26 (22.2)	115 (20.0)	0.88 (0.54, 1.42)
Missing	2 (1.7)	10 (1.7)	
Think you will get any type of injury today			
Disagree	88 (75.2)	355 (61.8)	1.00
Neither agree or disagree	11 (9.4)	81 (14.1)	1.83 (0.93, 3.57)
Agree	16 (13.7)	123 (21.4)	1.91 (1.08, 3.37)
Missing	2 (1.7)	15 (2.6)	
Think you will get a head injury today			
Disagree	95 (81.2)	423 (73.7)	1.00
Neither agree or disagree	6 (5.1)	65 (11.3)	2.43 (1.02, 5.78)
Agree	14 (12.0)	72 (12.5)	1.16 (0.62, 2.13)
Missing	2 (1.7)	14 (2.4)	
Think you will get a wrist injury today			
Disagree	87 (74.4)	379 (66.0)	1.00
Neither agree or disagree	8 (6.8)	70 (12.2)	2.01 (0.93, 4.33)
Agree	20 (17.1)	117 (20.4)	1.34 (0.79, 2.28)
Missing	2 (1.7)	8 (1.4)	
Use terrain parks			
Hardly ever	75 (64.1)	24 (4.2)	1.00
Half the time	28 (23.9)	88 (15.3)	9.82 (5.25, 18.37)
Most of the time	13 (11.1)	462 (80.5)	111.06 (54.18, 227.64)
Missing	1 (0.9)	0	
I plan to use the terrain park next time	51 (43.6)	528 (92.0)	88.74 (38.54, 204.30)
Missing	6 (5.1)	39 (6.8)	
Terrain parks are cool/challenging/fun^a^	41 (35.0)	428 (74.6)	5.43 (3.56, 8.30)
Terrain parks are for experienced skiers and snowboarders only^a^	69 (59.0)	214 (37.3)	0.41 (0.28, 0.62)
Terrain parks are riskier than the regular hill	88 (75.2)	354 (61.7)	0.53 (0.34, 0.83)
Terrain parks are too busy/crowded^a^	47 (40.2)	158 (27.5)	0.57 (0.38, 0.86)
Missing	0	1 (0.2)	
Terrain parks are the main reason I am here	9 (7.7)	362 (63.1)	20.49 (10.17, 41.30)
I get hurt in terrain parks^a^			
Never	40 (34.2)	57 (9.9)	1.00
Hardly Ever	34 (29.1)	346 (60.3)	7.14 (4.18, 12.21)
Half the time	4 (3.4)	105 (18.3)	18.42 (6.27, 54.09)
Most of the time	2 (1.7)	37 (6.4)	12.98 (2.96, 56.98)
Always	2 (1.7)	23 (4.0)	8.07 (1.80, 36.18)
Do not use them	33 (28.2)	3 (0.5)	0.06 (0.02, 0.22)
Missing	2 (1.7)	3 (0.5)	
Parents use terrain park			
No	95 (81.2)	416 (72.5)	1.00
Sometimes	8 (6.8)	46 (8.0)	1.31 (0.60, 2.87)
Yes	9 (7.7)	72 (12.5)	1.83 (0.88, 3.78)
Missing	5 (4.3)	40 (7.0)	
Siblings use terrain park^a^			
No	73 (62.4)	251 (43.7)	1.00
Sometimes	14 (12.0)	95 (16.6)	1.97 (1.06, 3.66)
Yes	25 (21.4)	201 (35.0)	2.34 (1.43, 3.82)
Missing	5 (4.3)	27 (4.7)	
Friends use terrain park			
No	18 (15.4)	19 (3.3)	1.00
Sometimes	46 (39.3)	42 (7.3)	0.86 (0.40, 1.87)
Yes	44 (37.6)	502 (87.5)	10.81 (5.29, 22.08)
Missing	9 (7.7)	11 (1.9)	
If my parents said I was not allowed I would go to the terrain park and risk getting caught^a^	35 (29.9)	408 (71.1)	5.98 (3.86, 9.25)
Missing	1 (0.9)	8 (1.4)	
If my friends decided not to use the terrain park, I would go where they went	76 (65.0)	327 (57.0)	0.80 (0.53, 1.22)
Missing	1 (0.9)	32 (5.6)	
I think people use the terrain park because friends use it	56 (47.9)	396 (69.0)	2.42 (1.62, 3.63)
I think people use the terrain park because all the good skiers and snowboarders use it^a^	79 (67.5)	337 (58.7)	0.68 (0.45, 1.04)
I think people use the terrain park to impress people	67 (57.3)	276 (48.1)	0.69 (0.46, 1.03)
I think terrain parks are more dangerous so you should wear a helmet			
Disagree	13 (11.1)	82 (14.3)	1.00
Neither agree or disagree	4 (3.4)	66 (11.5)	2.62 (0.81, 8.40)
Agree	99 (84.6)	423 (73.7)	0.68 (0.36, 1.27)
Missing	1 (0.9)	3 (0.5)	
I think terrain parks are used by my friends and I do not want to be left out^a^			
Disagree	65 (55.6)	321 (55.9)	1.00
Neither agree or disagree	32 (27.4)	116 (20.2)	0.73 (0.46, 1.18)
Agree	19 (16.2)	130 (22.6)	1.39 (0.80, 2.40)
Missing	1 (0.9)	7 (1.2)	
I think terrain parks are more dangerous than the regular hill			
Disagree	15 (12.8)	100 (17.4)	1.00
Neither agree or disagree	8 (6.8)	61 (10.6)	1.14 (0.46, 2.86)
Agree	94 (80.3)	409 (71.3)	0.65 (0.36, 1.17)
Missing	0	4 (0.7)	
Be aware in terrain parks to make terrain parks safer			
Disagree	11 (9.4)	67 (11.7)	1.00
Neither agree or disagree	3 (2.6)	35 (6.1)	1.92 (0.50, 7.32)
Agree	103 (88.0)	466 (81.2)	0.74 (0.38, 1.46)
Missing	0	6 (1.0)	
Go really fast to make terrain parks safer			
Disagree	74 (63.2)	312 (54.4)	1.00
Neither agree or disagree	20 (17.1)	97 (16.9)	1.15 (0.67, 1.98)
Agree	23 (19.7)	160 (27.9)	1.65 (1.00, 2.73)
Missing	0	5 (0.9)	
Slow down to make terrain parks safer			
Disagree	25 (21.4)	152 (26.5)	1.00
Neither agree or disagree	21 (17.9)	125 (21.8)	0.98 (0.52, 1.83)
Agree	71 (60.7)	292 (50.9)	0.68 (0.41, 1.11)
Missing	0	5 (0.9)	
Do not be scared of getting hurt to make terrain parks safer^a^			
Disagree	45 (38.5)	154 (26.8)	1.00
Neither agree or disagree	30 (25.6)	105 (18.3)	1.02 (0.61, 1.73)
Agree	42 (35.9)	311 (54.2)	2.16 (1.36, 3.44)
Missing	0	4 (0.7)	
Do not use dangerous features to make terrain parks safer			
Disagree	33 (28.2)	218 (38.0)	1.00
Neither agree or disagree	27 (23.1)	108 (18.8)	0.61 (0.35, 1.06)
Agree	57 (48.7)	240 (41.8)	0.64 (0.40, 1.02)
Missing	0	8 (1.4)	
Ski or snowboard within my ability to make terrain parks safer			
Disagree	14 (12.0)	83 (14.5)	1.00
Neither agree or disagree	4 (3.4)	53 (9.2)	2.23 (0.70, 7.15)
Agree	98 (83.8)	434 (75.6)	0.75 (0.41, 1.37)
Missing	1 (0.9)	4 (0.7)	
Take turns on features to make terrain parks safer			
Disagree	14 (12.0)	95 (16.6)	1.00
Neither agree or disagree	16 (13.7)	67 (11.7)	0.62 (0.28, 1.35)
Agree	86 (73.5)	406 (70.7)	0.70 (0.38, 1.28)
Missing	1 (0.9)	6 (1.0)	
Listen to music while skiing or snowboarding make terrain parks safer			
Disagree	61 (52.1)	233 (40.6)	1.00
Neither agree or disagree	31 (26.5)	125 (21.8)	1.06 (0.65, 1.71)
Agree	24 (20.5)	210 (36.6)	2.29 (1.38, 3.81)
Missing	1 (0.9)	6 (1.0)	
Fewer people in the park at one time make terrain parks safer			
Disagree	27 (23.1)	195 (34.0)	1.00
Neither agree or disagree	27 (23.1)	107 (18.6)	0.55 (0.31, 0.98)
Agree	62 (53.0)	265 (46.2)	0.59 (0.36, 0.96)
Missing	1 (0.9)	7 (1.2)	

^a^Variables included in the logistic regression model

**Table 3:** Baseline characteristics for those video recording and not video recording while skiing or snowboarding today (*N* = 724)

	Not recording myself or others while skiing or snowboarding today *n* = 474 (%)	Recording myself or others while skiing or snowboarding today *n* = 250 (%)	OR (95% CI)
Age (years)	14.8 (1.5)	14.9 (1.5)	1.05 (0.94, 1.16)
Male	369 (77.8)	224 (89.6)	2.50 (1.57, 3.99)
Missing	2 (0.4)	1 (0.4)	
Snowboarders	376 (79.3)	218 (87.2)	1.80 (1.15, 2.82)
Missing	8 (1.7)	3 (1.2)	
Ability			
Beginner	62 (13.1)	18 (7.2)	1.00
Intermediate	214 (45.1)	69 (27.6)	1.11 (0.62, 2.01)
Advanced	148 (31.2)	90 (36.0)	2.09 (1.17, 3.77)
Expert	40 (8.4)	62 (24.8)	5.34 (2.76, 10.31)
Missing	10 (2.1)	11 (4.4)	
Previous ski/snowboard injury that required a doctor^a^	99 (20.9)	87 (34.8)	2.13 (1.50, 3.03)
Missing	52 (11.0)	30 (12.0)	
Skiing or snowboarding alone	45 (9.5)	30 (12.0)	1.29 (0.79, 2.11)
Missing	2 (0.4)	0	
Skiing or snowboarding with friends today^a^	403 (85.0)	231 (92.4)	2.29 (1.30, 4.06)
Missing	7 (1.5)	3 (1.2)	
Skiing or snowboarding with parents today	43 (9.1)	20 (8.0)	0.86 (0.49, 1.49)
Missing	15 (3.2)	4 (1.6)	
Skiing or snowboarding with siblings today	101 (21.3)	47 (18.8)	0.84 (0.57, 1.24)
Missing	10 (2.1)	2 (0.8)	
Think you will get any injury today			
Disagree	315 (66.5)	149 (59.6)	1.00
Neither agree or disagree	65 (13.7)	34 (13.6)	1.11 (0.70, 1.75)
Agree	84 (17.7)	62 (24.8)	1.56 (1.07, 2.29)
Missing	10 (2.1)	5 (2.0)	
Think you will get a head injury today^a^			
Disagree	378 (79.7)	166 (66.4)	1.00
Neither agree or disagree	38 (8.0)	38 (15.2)	2.28 (1.40, 3.70)
Agree	48 (10.1)	42 (16.8)	1.99 (1.27, 3.13)
Missing	10 (2.1)	4 (1.6)	
Think you will get a wrist injury today			
Disagree	339 (71.5)	150 (60.0)	1.00
Neither agree	50 (10.5)	34 (13.6)	1.54 (0.95, 2.47)
Agree	78 (16.5)	64 (25.6)	1.85 (1.27, 2.72)
Missing	7 (1.5)	2 (0.8)	
I plan to record myself or others next time^a^	151 (31.9)	200 (80.0)	13.93 (9.01, 21.53)
Missing	18 (3.8)	21 (8.4)	
I record myself while skiing/snowboarding because I am confident in my skills^a^	207 (43.7)	183 (73.2)	3.43 (2.46, 4.79)
Missing	7 (1.5)	0	
Friends record videos while skiing/snowboarding^a^			
No	103 (21.7)	12 (4.8)	1.00
Sometimes	169 (35.7)	46 (18.4)	2.34 (1.18, 4.62)
Yes	163 (34.4)	174 (69.6)	9.16 (4.86, 17.29)
Missing	39 (8.2)	18 (7.2)	
Siblings record videos while skiing/snowboarding^b^			
No	337 (71.1)	140 (56.0)	1.00
Sometimes	69 (14.6)	32 (12.8)	1.12 (0.70, 1.77)
Yes	50 (10.5)	58 (23.2)	2.79 (1.82, 4.28)
Missing	18 (3.8)	20 (8.0)	
Parents record videos while skiing/snowboarding^b^			
No	394 (83.1)	173 (69.2)	1.00
Sometimes	29 (6.1)	22 (8.8)	1.73 (0.97, 3.09)
Yes	33 (7.0)	33 (13.2)	2.28 (1.36, 3.81)
Missing	18 (3.8)	22 (8.8)	
I record myself or others while skiing/snowboarding			
Less than half the time	355 (74.9)	54 (21.6)	1.00
Half the time	92 (19.4)	109 (43.6)	7.79 (5.23, 11.60)
More than half the time	26 (5.5)	87 (34.8)	22.00 (13.03, 37.12)
Missing	1 (0.2)	0	
I think recording makes me try harder and improve my tricks^a^			
Disagree	102 (21.5)	49 (19.6)	1.00
Neither agree or disagree	68 (14.3)	18 (7.2)	0.55 (0.30, 1.03)
Agree	302 (63.7)	181 (72.4)	1.25 (0.85, 1.84)
Missing	2 (0.4)	2 (0.8)	
I think recording makes me nervous and can increase my risk of getting hurt			
Disagree	182 (38.4)	132 (52.8)	1.00
Neither agree or disagree	123 (25.9)	48 (19.2)	0.54 (0.36, 0.80)
Agree	164 (34.6)	66 (26.4)	0.55 (0.39, 0.80)
Missing	5 (1.1)	4 (1.6)	

^a^Variables included in the logistic regression model

^b^Parent and siblings were combined to ‘any family member’ in the logistic regression model

**Table 4:** Prevalence of high-risk behaviours (proportion; 95% CI)

Listening to music							
Overall (*n* = 723)	Males (*n* = 591)	Females (*n* = 129)	13–14 (*n* = 331)	15–16 (*n* = 271)	17–18 (*n* = 121)	Snowboard (*n* = 597)	Ski (*n* = 116)
36.9 (33.4, 40.5)	39.8 (35.8, 43.7)	23.2 (15.9, 30.6)	28.4 (23.5, 33.3)	43.5 (37.6, 49.5)	45.5 (36.5, 54.5)	39.0 (35.1, 43.0)	25.0 (17.0, 33.0)
Using the terrain park							
Overall (*n* = 691)	Males (*n* = 564)	Females (*n* = 124)	13–14 (*n* = 319)	15–16 (*n* = 256)	17–18 (*n* = 116)	Snowboard (*n* = 564)	Ski (*n* = 121)
83.1 (80.3, 85.9)	88.7 (86.0, 91.3)	57.3 (48.4, 66.1)	85.0 (81.0, 88.9)	82.4 (77.7, 87.1)	79.3 (71.8, 86.8)	85.6 (82.7, 88.5)	70.0 (61.7, 78.5)
Video recording							
Overall (*n* = 724)	Males (*n* = 593)	Females (*n* = 128)	13–14 (*n* = 332)	15–16 (*n* = 269)	17–18 (*n* = 123)	Snowboard (*n* = 594)	Ski (*n* = 119)
34.5 (31.1, 38.0)	37.8 (33.9, 41.7)	19.5 (12.6, 26.5)	33.1 (28.0, 38.2)	34.9 (29.2, 40.7)	37.4 (28.7, 46.1)	36.7 (32.8, 40.6)	24.3 (16.5, 32.2)

### Music

Overall, 267 (36.9%) of youth reported listening to music that day (Table 1). Of those 267 youth, the majority (*N* = 207) use one method to listen to music (103 one earbud only, 86 two ear buds only, and 18 a helmet with built in speakers only). Compared to those not listening to music today, those who reported listening to music today were significantly older. They were also more likely to be male, snowboarding, had sustained a previous skiing or snowboarding injury, were skiing or snowboarding alone or with friends, or reported their skill level as advanced or expert compared with beginner.

Results of the multivariable logistic regression model showed youth had significantly higher odds of listening to music on a personal device if they reported that they planned to listen to music next time (OR 19.13; 95% CI: 10.62, 34.44) (Table 5). Youth who were skiing or snowboarding alone had significantly higher odds of listening to music (OR 2.33; 95% CI: 1.10, 4.95) along with those who thought listening to music makes skiing or snowboarding more exciting or fun or makes them feel more confident (OR 2.30; 95% CI: 1.31, 4.05). Youth had significantly lower odds of listening to music if they considered themselves advanced (OR 0.46; 95% CI: 0.22, 0.93), intermediate (OR 0.36; 95% CI: 0.18, 0.73), or beginner (OR 0.36; 95% CI: 0.13, 0.98) compared to those who considered themselves experts. Youth who agreed that listening to music makes it more difficult to hear or talk to others had significantly lower odds of listening to music than those who disagreed with this statement (OR: 0.35; 95% CI: 0.18, 0.65).

**Table 5:** Those who were versus those who were not listening to music (*N* = 625; OR (95% CI))

	OR (95% CI)
I plan to listen to music next time	19.13 (10.62, 34.44)
Makes skiing or snowboarding more exciting or fun or makes them more confident	2.30 (1.31, 4.05)
Ability	
Beginner	0.36 (0.13, 0.98)
Intermediate	0.36 (0.18, 0.73)
Advanced	0.46 (0.22, 0.93)
Expert	1.00
Skiing or snowboarding alone	2.33 (1.10, 4.95)
Music makes me a better skier or snowboarder	
Neither agree or disagree	0.94 (0.43, 2.03)
Agree	1.65 (0.89, 3.07)
Disagree	1.00
Listening to music is safe if you use one ear bud	
Neither agree or disagree	1.67 (0.72, 3.87)
Agree	1.68 (0.91, 3.10)
Disagree	1.00
Listening to music makes me more careful	
Neither agree or disagree	1.33 (0.69, 2.54)
Agree	0.80 (0.45, 1.43)
Disagree	1.00
Listening to music is distracting	
Neither agree or disagree	0.54 (0.27, 1.10)
Agree	0.59 (0.33, 1.07)
Disagree	1.00
Listening to music makes it harder to hear/talk to people	
Neither agree or disagree	0.46 (0.19, 1.13)
Agree	0.35 (0.18, 0.65)
Disagree	1.00
I don’t like the music playing overhead	0.97 (0.59, 1.60)
Family listens to music while skiing or snowboarding	
Sometimes	1.48 (0.76, 2.87)
Yes	1.84 (1.02, 3.32)
No	1.00

### Terrain parks

There were 451 (83.1%) youth who reported using the TP. TP users were significantly more likely to be male, snowboarding, consider themselves intermediate, advanced, or expert skiers or snowboarders compared to beginners, skiing or snowboarding with friends, or have sustained a previous skiing or snowboarding injury (Table 2).

The odds of using the TP significantly decreased with each increasing year of age (OR: 0.70; 95% CI: 0.57, 0.86) or if youth believed that TPs were too busy or crowded (OR: 0.31; 95% CI: 0.16, 0.62) (Table 6). Youth who thought that TPs were for experienced skiers and snowboarders only (OR: 0.32; 95% CI: 0.16, 0.64) or that people use the TP because all the good skiers and snowboarders use it (OR: 0.45; 95% CI: 0.22, 0.90) were significantly less likely to use the TP than those who did not agree with those statements. Youth had significantly higher odds of using the TP if they reported their ability as intermediate (OR: 2.85; 95% CI: 1.17, 6.95) or advanced (OR: 3.78; 95% CI: 1.41, 10.19) compared with beginner. Youth also had significantly higher odds of using the TP if they had a previous ski or snowboard injury that resulted in a physician visit (OR: 3.05; 95% CI: 1.18, 7.90) or got injured in TPs at least of half the time (OR: 5.85; 95% CI: 2.21, 15.50) compared with less than half of the time. Youth had significantly higher odds of using the TP if they were skiing or snowboarding with friends that day (OR: 3.96; 95% CI: 1.71, 9.20) or their siblings use the TP when skiing or snowboarding (OR: 4.94; 95% CI: 2.48, 9.85). They also used TPs if they thought TPs were cool, challenging, or fun (OR: 5.84; 95% CI: 2.85, 11.96). Youth who believed that not being afraid of getting hurt made TPs safer (OR: 2.07; 95% CI: 1.04, 4.13) also had significantly higher odds of using the TP that day.

**Table 6:** Those who were versus those who were not using terrain parks (*N* = 542; OR: 95% CI))

	OR (95% CI)
Age (Years)	0.70 (0.57, 0.86)
Sex	4.18 (2.01, 8.70)
Snowboarding today	1.40 (0.64, 3.04)
Ability	
Beginner	1.00
Intermediate	2.85 (1.17, 6.95)
Advanced/Expert	3.78 (1.41, 10.19)
Previous ski/snowboard injury that required a doctor	3.05 (1.18, 7.90)
Skiing or snowboarding with friends today	3.96 (1.71, 9.20)
Terrain parks are cool/challenging/fun	5.84 (2.85, 11.96)
Terrain parks are for experienced skiers and snowboarders only	0.32 (0.16, 0.64)
Terrain parks are too busy/crowded	0.31 (0.16, 0.62)
I think terrain parks are used by my friends and I don’t want to be left out	0.88 (0.45, 1.72)
I get hurt in terrain parks at least half the time	5.85 (2.21, 15.50)
Do not be scared of getting hurt to make terrain parks safer	2.07 (1.04, 4.13)
Siblings use the terrain park when skiing/snowboarding	4.94 (2.48, 9.85)
If my parents said I wasn’t allowed I would go to the terrain park and risk getting caught	2.22 (1.10, 4.49)
I think people use the terrain park because all the good skiers and snowboarders use it	0.45 (0.22, 0.90)

### Video recording themselves or others

Overall, 250 (34.5%) of youth reported recording themselves or others on the day of survey completion: 105 typically used only one device, 85 used two devices, 35 used three devices, 22 used all four recording devices (cell phone, digital camera, GoPro, or helmet mounted camera), and 3 did not indicate what type of device they use. The most common device used was a helmet mounted camera (46.0%), followed by digital camera (45.2%). Youth recording themselves or others on that day were significantly more likely to be male, snowboarding**,** consider themselves advanced or expert skiers and snowboarders, to be skiing or snowboarding with friends, or to have sustained a previous ski/snowboard injury that required a doctor (Table 3).

Youth had significantly lower odds of video recording that day if they reported that their friends do not record videos while skiing or snowboarding (OR: 0.36, 95% CI: 0.16, 0.80) or only sometimes record videos while skiing or snowboarding (OR: 0.41, 95% CI: 0.24, 0.69) compared to those whose friends do record (Table 7). Compared to those who disagreed with the statement “I think I will get a head injury today”, youth who agreed (OR: 2.05, 95% CI: 1.09, 3.83) or neither agreed or disagreed (OR: 2.93, 95% CI: 1.41, 6.08) had significantly higher odds of video recording. Youth had significantly higher odds of video recording if they reported that they plan to video record next time (OR: 8.09, 95% CI: 4.67, 14.01). Youth had significantly higher odds of video recording if they disagreed (OR: 5.16, 95% CI: 1.92, 13.89) or agreed (OR: 3.34, 95% CI: 1.38, 8.08) with the statement “I think recording makes me try harder and improve my tricks” compared to those who neither agreed nor disagreed. Youth had significantly higher odds of recording themselves or others if they were skiing or snowboarding with friends that day (OR: 3.65, 95% CI: 1.45, 9.18).

**Table 7:** Those who were versus those who were not recording themselves or others (*N* = 541; OR: 95% CI))

	OR (95% CI)
Previous ski/snowboard injury that required a doctor	0.90 (0.56, 1.43)
Skiing or snowboarding with friends today	3.65 (1.45, 9.18)
Think you will get a head injury today	
Disagree	1.00
Neither agree or disagree	2.93 (1.41, 6.08)
Agree	2.05 (1.09, 3.83)
I plan to record myself or others next time	8.09 (4.67, 14.01)
I think recording makes me try harder and improve my tricks	
Disagree	5.16 (1.92, 13.89)
Neither Agree or Disagree	1.00
Agree	3.34 (1.38, 8.08)
Family records videos while skiing/snowboarding	
No	0.56 (0.31, 1.01)
Sometimes	0.65 (0.29, 1.43)
Yes	1.00
Friends record videos while skiing/snowboarding	
No	0.36 (0.16, 0.80)
Sometimes	0.41 (0.24, 0.69)
Yes	1.00
I record myself while skiing/snowboarding because I am confident in my skills	1.08 (0.65, 1.81)

## Discussion

The sports of skiing and snowboarding are continuously evolving and there have been changes both to equipment and behaviours performed during participation. This is the first study to examine the prevalence and predictors of three relatively new risk behaviours: listening to music, using video recorders, and using the TP.

Consistent with the TPB, there were common predictors of youth engaging in the behaviours studied. Attitudes towards the behaviours were important predictors. Those who believed the behaviours made snowboarding and skiing more fun or challenging not only engaged in the behaviours at the time of study but intended to do so again in the future. Previous research also has found that risky decisions during play are often motivated by youth seeking to enhance their fun [29]. Moreover, youth have been shown to underestimate injury risk (vulnerability, severity) when they observe peers who are emphasizing fun by smiling while risk taking [30]. Skiing or snowboarding with peers was associated with using the TP in this study, and the social-based focus on fun may contribute to explain this finding. Subjective norms communicated by peers and siblings also came into play in the present study. Consistent with past studies of youth relationships, having significant others (e.g., friends or siblings) engaging in risk behaviours predicted both youth doing so and their planning to continue the practice in the future [29, 39]. Importantly, efforts to modify youths’ perceived behavioural norms have proven effective to reduce their risk behaviours [40–43], suggesting that this may be a strategy that can be applied to snowboarding and skiing.

Finally, as the TPB predicts, the importance of perceived behavioural control was indicated by the fact that those concerned about safety and their ability to avoid injury did not engage in the behaviours of listening to music or video recording. Similarly, those who believed that not being afraid of getting hurt made the TP safer were more likely to use the park. Past research findings also have shown that beliefs about preventability of injury, injury severity, and control over one’s safety are important determinants of youth decisions to either engage in or avoid risky behaviours during play that can elevate risk of injury [26, 44, 45]. For example, when youth are concerned about the potential severity of an injury, this is associated with reduced risk taking [26]. Thus, using a social marketing fear appeals approach that emphasizes consequences of injury severity may be another intervention strategy that can be applied. Research has shown that focusing on social consequences or losses that are linked to risk behaviours (e.g., missing out on parties due to hospitalization from injury) is particularly effective when youth are the target audience [46].

Interestingly, using the TP was associated with having experienced previous injuries during the sport that resulted in visiting a doctor. Although one might expect that this experience would result in greater beliefs about injury severity and, therefore, avoidance of the park, these youth actually rated themselves as having a high level of experience in the sport. Past research has shown that experience leads youth to underestimate injury risk and severity, with greater risk taking as youth accumulate experience in a sport [47]. One possible way to counteract these effects of experience could be by exposing these youth to injury stories created by youth with similar experiences who were injured. Morrongiello and her colleagues applied this approach with elementary school children and found that it was effective to reduce optimism bias and risk taking on playgrounds [30].

In sum, overall, the pattern of these diverse findings suggests that the TPB is a useful tool for understanding youth behaviour in skiing and snowboarding situations and guiding strategies for intervention.

### Limitations

During questionnaire development using the TP, listening to music, and video recording were positively correlated to sensation seeking and risk taking, however the correlations were not very strong. This was most evident for sensation seeking. The sensation seeking tool was developed in the late 1980s and perhaps some of the examples of sensation seeking are no longer relevant to today’s youth. Additionally, access to YouTube and other video streaming sites may cause youth to become desensitized to sensation seeking or high-risk behaviours [48]. In fact, one of the questions in the sensation seeking questionnaires is related to the desire to ski or snowboard quickly and this might be over represented among this sampled population. Additionally, the data are self-reported and it is possible that the participants were not truthful in their responses. However, the survey questions were not particularly personal or sensitive, reducing the chances of social desirability bias. Youth were instructed to only complete the survey once but there may be instances where someone completed it more than once. Two of the three RAs were at every data collection session and recognized some youth who had already completed the survey and prevented them from doing it again. Also, youth were asked if they had previously completed the survey and were told upon completion that they could only complete it once. Future efforts could attempt to determine ways to prevent youth from repeated participation during the same day; possibly by placing a stamp on their hand once they do participate. There may have been survey fatigue given that some questions were incomplete and some youth were excluded because they provided contradictory responses. The data were collected several years ago and the findings may not be generalizable to the behaviours of current skiing and snowboarding youth. There were a large number of comparisons and statistical tests performed which may have increased the risk of finding spurious associations. Finally, the baseline characteristics of those who provided and did not provide complete results were similar for video recording and using the TP; however, there was different proportions of ability in those who reported complete data for listening to music.

## Conclusions and future directions

We identified some common predictors of engaging in high-risk behaviours while skiing or snowboarding including planning to next time, friends or siblings engaging in the behaviours, or believing that the behaviours were fun or challenging. Deterrents of engaging in the behaviours included safety concerns. Although not measured in this study, those who engage in more high-risk behaviours may have an increased risk of injury. Future directions include determining the nuances of the risky behaviour and the risk of injury. For instance, the hands-free GoPro may be less risky or a GoPro may be more risky if the youth is more inclined to ski or snowboard in a riskier manner in an effort to capture more extreme footage. Expanding our measures to directly tap youth beliefs about some psychosocial measures, such as perceptions of vulnerability and injury severity, would provide valuable information to determine if these vary for skiers and snowboarders in ways that need to be considered when planning interventions to reduce risk taking. Moreover, tracking youth over time would enable us to relate measures to injury outcomes, which may provide unique insights into psychosocial determinants that differentiate youth who are and are not injured. Future work is needed to confirm this link between psychosocial predictors of high-risk behaviours and injury.

In conclusion, the common themes among the three behaviours indicate that injury prevention programs may not have to be specific to each behaviour individually. Rather, injury prevention programs may be effective if they can target common determinants to reduce risk behaviours among youth while skiing and snowboarding.

## Supplementary information

**Additional file 1.** Final Questionnaire: AdditionalFile_Questionnaire_06Apr2020

## Data Availability

The datasets generated and/or analysed during the current study are not publically available because the individual participants did not consent to their individual data being made publically available. Requests to access the dataset should be directed to Shelly Rempel-Rossum at Shelly.rempel-rossum@umanitoba.ca

## Abbreviations

OR: Odds Ratio
RA: Research Assistant
TP: Terrain Park
TPB: Theory of Planned Behaviour
