# Supplementary material for: Understanding youths’ attitudes and practices regarding listening to music, video recording and terrain park use while skiing and snowboarding
Source: BMC Pediatr. 2020 Aug 19;20:389. doi: 10.1186/s12887-020-02292-6 (PMC7437043; doi:10.1186/s12887-020-02292-6)
Supplement: Supplementary file 1 — Additional file 1. Final Questionnaire: AdditionalFile_Questionnaire_06Apr2020 [file 12887_2020_2292_MOESM1_ESM.docx]

**Age:** ____ yrs. **Sex:**
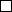
 Male
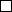
 Female

**Sport:**
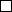
 Ski
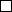
 Snowboard

**Ability:**
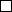
 Beginner
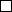
 Intermediate
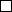
 Advanced
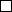
 Expert

**Previous ski/snowboard injury and went to a doctor:**
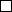
 Yes
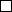
 No

**Today**, I’m skiing/snowboarding:

By myself
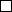
 Yes
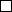
 No

Friends
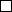
 Yes
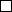
 No Wear helmets?
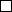
 Yes
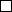
 No
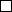
 Some

Parents
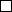
 Yes
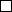
 No Wear helmets?
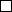
 Yes
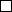
 No
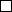
 Some

Brothers/sisters
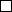
 Yes
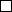
 No Wear helmets?
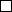
 Yes
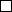
 No
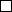
 Some

Other ______________
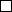
 Yes
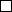
 No Wear helmets?
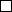
 Yes
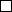
 No
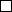
 Some

On a scale of 1-7, I think I will get hurt **today** while skiing/snowboarding?

DISAGREE: 1 = Totally, 2 = Somewhat, 3 = Little

NEITHER Agree or Disagree: 4

AGREE: 5 = Little, 6 = Somewhat, 7 = Totally

Any injury____

Head injury____

Wrist injury____

**TERRAIN PARKS**

I use the terrain parks when skiing/snowboarding:


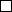
 Never
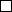
 Hardly ever
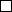
 Half the time
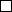
 Most of the time
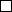
 Always

I’m using the terrain park today:
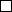
 Yes
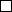
 No

I plan to use the terrain park next time I go skiing/snowboarding:
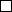
 Yes
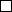
 No

Check all that apply- Terrain parks are:


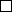
 Cool
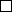
 Challenging
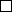
 Fun


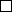
 For experienced riders only


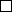
 Riskier than the regular hill


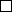
 Too busy/crowded


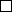
 The main reason I am here

On a scale of 1-7, I think terrain parks are:

DISAGREE: 1 = Totally, 2 = Somewhat, 3 = Little,

NEITHER Agree or Disagree: 4

AGREE: 5 = Little, 6 = Somewhat, 7 = Totally

More dangerous than the regular hill ____

More dangerous so you should always wear a helmet in the park ____

Used by my friends and I don’t want to be left out ____

I get hurt in the terrain parks:


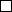
 Never
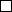
 Hardly ever
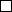
 Half the time
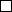
 Most of the time
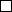
 Always
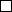
 Don’t use them

On scale of 1-7, the following make terrain parks safer:

DISAGREE: 1 = Totally, 2 = Somewhat, 3 = Little

NEITHER Agree or Disagree: 4

AGREE: 5 = Little, 6 = Somewhat, 7 = Totally

Be aware of other people ____

Go really fast ____

Slow down ____

Don’t be scared of getting hurt ____

Don’t use the dangerous features ____

Ride within my ability ____

Take turns on the features ____

Listen to music when riding ____

Fewer people in the park at one time ____

Do these people use the terrain park when skiing/snowboarding?

Parents
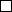
 Yes
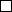
 Sometimes
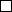
 No
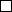
 Don’t know
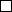
 Don’t ski/snowboard

Brothers/sisters
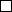
 Yes
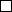
 Sometimes
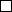
 No
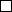
 Don’t know
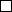
 Don’t ski/snowboard

Friends
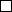
 Yes
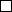
 Sometimes
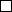
 No
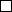
 Don’t know
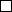
 Don’t ski/snowboard

If my parents said I wasn’t allowed to use the terrain park, I would go anyways and risk get caught:
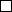
 Yes
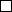
 No

If my friends decided not to use the terrain park, I would go where they went:
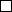
 Yes
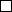
 No

Check all that apply- I think people use the terrain park because:

Friends use it
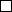


All the good riders use it
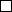


Impress people
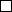


**LISTENING TO MUSIC**

I listen to music on my iPod/phone while skiing/snowboarding:


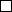
 Never
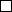
 Hardly ever
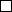
 Half the time
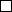
 Most of the time
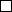
 Always

Today, I’m listening to music on my iPod/phone while riding:


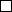
 Yes
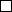
 No
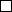
 Sometimes
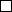
 Regular hill only
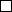
 Terrain park only

I plan to listen to music next time I go skiing/snowboarding:
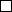
 Yes
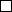
 No

Check all that apply – While skiing/snowboarding, I listen to music through:


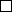
 Helmet with built in speakers


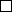
 Two ear buds


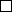
 One ear bud only


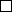
 Don’t listen to music on iPod/phone

On a scale of 1-7, I think listening to music while riding:

DISAGREE: 1 = Totally, 2 = Somewhat, 3 = Little,

NEITHER Agree or Disagree: 4

AGREE: 5 = Little, 6 = Somewhat, 7 = Totally

Fun or relaxing ____

Makes me a better rider ____

More likely to hurt me or others ____

Is safe if I use only one ear bud ____

Makes me more careful ____

Is distracting ____

Makes it harder to hear/talk to people ____

Check all that apply - I listen to music while riding because:


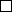
 I want to listen to my own music


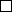
 I don’t like the music playing overhead


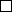
 Makes riding more exciting/fun


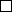
 Makes me more aware of my surroundings


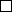
 I like being in my own world


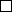
 It makes me more confident

Do these people listen to music when riding?

Parents
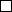
 Yes
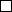
 Sometimes No Don’t know Don’t ski/snowboard

Brothers/sisters Yes Sometimes No Don’t know Don’t ski/snowboard

Friends Yes Sometimes No Don’t know Don’t ski/snowboard

**VIDEO RECORDING**

I record myself or others while skiing/snowboarding:

Never Hardly ever Half the time Most of the time Always

Today, I’m recording myself or others while riding:

Yes No Regular hill only Terrain park only

I plan to record myself or others next time I go skiing/snowboarding: Yes No

Check all that apply – I record skiing/snowboarding using a:

Cell phone

Digital Camera

GoPro

Camera/helmet mount

On a scale of 1-7, I think recording while riding:

DISAGREE: 1 = Totally, 2 = Somewhat, 3 = Little,

NEITHER Agree or Disagree: 4

AGREE: 5 = Little, 6 = Somewhat, 7 = Totally

Makes me try harder and improve my tricks ____

Makes me nervous and can increase my risk of getting hurt ____

Do these people record videos while skiing/snowboarding?

Parents Yes Sometimes No Don’t know Don’t ski/snowboard

Brothers/sisters Yes Sometimes No Don’t know Don’t ski/snowboard

Friends Yes Sometimes No Don’t know Don’t ski/snowboard

I record myself while skiing/snowboarding because I am confident in my skills:

Yes No
